# Advances in DNA damage response inhibitors in colorectal cancer therapy: Advances in DNA damage response inhibitors in colorectal cancer

**DOI:** 10.3724/abbs.2023278

**Published:** 2023-12-19

**Authors:** Yue Yu, Hang Jia, Tianshuai Zhang, Wei Zhang

**Affiliations:** Department of Colorectal Surgery the First Affiliated Hospital Naval Medical University Shanghai 200433 China

**Keywords:** DNA damage response, colorectal cancer, targeted therapy, DDR inhibitor

## Abstract

One potential cause of cancer is genomic instability that arises in normal cells due to years of DNA damage in the body. The clinical application of radiotherapy and cytotoxic drugs to treat cancer is based on the principle of damaging the DNA of cancer cells. However, the benefits of these treatments also have negative effects on normal tissue. While there have been notable advancements in molecular-driven therapy and immunotherapy for colorectal cancer (CRC), a considerable portion of patients with advanced CRC do not experience any benefits from these treatments, leading to a poor prognosis. In recent years, targeted therapy aimed at suppressing the DNA damage response (DDR) in cancer cells has emerged as a potential treatment option for CRC patients, offering them more choices for treatment. Currently, the integration of DDR and clinical intervention remains in the exploratory phase. This review primarily elucidates the fundamental principles of DDR inhibitors, provides an overview of their current clinical application status in CRC, and discusses the advancements as well as limitations observed in relevant studies.

## Introduction

**Figure 1:**
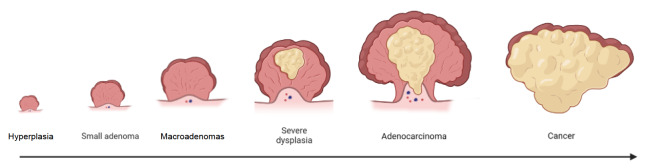
Figure 1 The progression of colorectal cancer The development of colorectal cancer from proliferative polyps in the intestine is described.

Colorectal cancer (CRC) constitutes approximately 10% of all malignancies and ranks as the second leading cause of cancer-related mortality [1]. The pathological progression originates from hyperplasia, which subsequently progresses into benign adenomatous polyps. Over a prolonged period of time, dysplasia develops until it becomes an adenoma. Ultimately, this adenoma undergoes complete transformation into cancer and may metastasize ( Figure 1).

Microsatellites are repetitive DNA sequences that exhibit higher mutation rates compared to other genomic regions. Microsatellite instability (MSI) is the phenotypic manifestation of deficient DNA mismatch repair (dMMR), which arises from mutational inactivation of genes involved in DNA mismatch repair, including MLH1, MSH2, MSH3, MSH6, and PMS2 [2]. According to MMR status, CRC can be divided into two major subtypes: microsatellite instability (MSI) and microsatellite stability (MSS) [3].

The DNA within cells is subject to daily influences from a range of internal factors and the external environment, resulting in DNA damage. Under normal circumstances, this damage can be effectively repaired through various intracellular pathways, thereby ensuring proper cell proliferation and apoptosis. The intracellular DNA damage response (DDR) pathway represents a sophisticated protein network system that plays a crucial role in maintaining genome integrity. Dysfunctions in the intracellular DDR have been implicated in numerous diseases including tumors, neurodegenerative disorders, and immune deficiencies [4]. The hallmark of tumor cells lies in their genomic instability, primarily characterized by alterations in chromosome number and structure, as well as MSI [5]. Therefore, DDR has emerged as a pivotal strategy for the development of anticancer drugs. Targeted inhibitors primarily exert their effects on protein kinases or selectively induce tumor cell death through synergistic lethal mechanisms. Among various DDR pathway inhibitors, poly ADP-ribose polymerase (PARP) inhibitors have garnered extensive research attention and are currently approved by the Food and Drug Administration (FDA), including olaparib, rucaparib, and niraparib, for clinical management of several advanced malignancies [ 6‒ 8].

Despite significant advancements in molecular and immunotherapeutic approaches, the prognosis for advanced disease remains unfavorable. Alterations in DDR pathways are emerging as novel therapeutic targets for various cancer types. This review paper provides an overview of the fundamental principles, current clinical applications, and recent research progress pertaining to DDR inhibitors.

## Mechanisms of DDR in Regulating CRC

### Proteins in DDR are the target of DDR inhibitors

Humans have developed a diverse array of mechanisms to effectively respond to DNA damage events. The DNA damage response encompasses a wide range of signaling pathways and enzymatic activities occurring within and between cells, triggered by the induction and examination of DNA damage [9]. These stimuli elicit biological responses, encompassing cell cycle arrest, regulation of DNA replication, and repair or encapsulation of DNA damage [10]. In the event of DNA repair failure and acquisition of stable genes, DDRs can also play a role in subsequent cell fate determinations, such as facilitating programmed cell death or senescence, which may be influenced by immune system factors to varying extents [11]. Recently conducted analysis indicates that DDRs encompass a minimum of 450 proteins. The efficacy of targeting DDRs relies on the specific inhibition of DNA damage response or the particular stage of the cell cycle in which cancer cells reside [12]. Different forms of DNA damage response are mediated by distinct repair mechanisms and signaling pathways. In the absence of optimal or intrinsic repair pathways, alternative DDR pathways may be activated to compensate [13].

### Five major DNA repair pathways in human cells

DNA single strand breaks (SSBs) are the most prevalent form of DNA damage, occurring at an average rate of over 20,000 instances per cell per day. These lesions are effectively repaired through the mechanism of base excision repair (BER) [14]. In cellular DNA repair mechanisms, two primary forms are involved in addressing double-strand breaks (DSBs): homologous recombination repair (HR) and nonhomologous end-joining repair (NHEJ) [15]. In recent years, there has been significant progress in the comprehension of alternative end-joining (A-EJ), not only as an alternative pathway to classical end-joining (c-NHEJ) but also as a potential competitor for DSB repair alongside c-NHEJ [16]. The most genotoxic form of DNA damage is represented by DSBs, as they exert an impact on the precise segregation of chromosomes during cellular division [17]. The HR pathway represents a relatively precise and efficient mechanism for DNA repair, contingent upon the availability of intact sister chromatids. In contrast, the NHEJ pathway operates independently of sister chromatid presence [18]. Although NHEJ is a proficient mechanism, it exhibits reduced fidelity and has the potential to induce DNA rearrangements. Nucleotide excision repair (NER), a prominent pathway for addressing ultraviolet (UV)-induced DNA damage, specifically targets modified nucleotides that distort the double helix structure. The mismatch repair (MMR) pathway effectively rectifies replication errors encompassing mismatched base pairing as well as nucleotide insertion and deletion [19]. Another prevalent occurrence during replication is the incorporation of ribonucleotides, which have a higher propensity to induce DNA strand breaks compared to deoxynucleotides due to the heightened susceptibility of ribonuclease towards hydrolysis [20].

Understanding the fundamental principle of DDR elucidates the rationale behind its abundance as a target for anticancer therapeutics. In contrast to normal cells, DDR confers protection to cancer cell DNA, facilitating their relentless division and proliferation. Consequently, targeting DDRs has emerged as a promising avenue for developing novel agents in targeted cancer therapy research. Notably, in rectal cancer treatment, radiotherapy has gained prominence due to its ability to induce DSBs. However, the clinical challenge lies in overcoming radioresistance observed in numerous patients with limited tumor regression. Therefore, there is an urgent need to combine DDR inhibitors with radiotherapy for effective management of rectal cancer.

### Mechanisms of DDR

By triggering a variety of posttranslational modifications in proteins and facilitating the assembly of protein complexes, DDR signaling proteins enhance and diversify intracellular damage signals while coordinating the most appropriate cellular responses. These responses include transcriptional changes, activation of cell cycle checkpoints, alternative splicing, participation in DNA repair processes, and under conditions of overwhelming damage, initiation of cellular senescence or apoptosis pathways [21]. Replication stress refers to the decoupling of DNA polymerase from replisome helicase activity [ 22, 23]. Consequently, the replication fork generates an elongated single-stranded DNA (ssDNA), which in turn triggers binding by replication protein A (RPA) and initiates a DDR primarily regulated by the ataxia telangiectasia and Rad3-related (ATR) kinase [24].

The ATR pathway safeguards against replication fork breakdown and DSB generation through diverse mechanisms, including activation of its effector kinase checkpoint kinase 1 (CHK1) under conditions of replication stress [ 25, 26]. The coordination of ribonucleotide reductase M2 (RRM2) is also facilitated by ATR [20]. This mechanism aids in preventing excessive accumulation of ssDNA, thereby mitigating the risk of RPA depletion.

The activity of DNA-dependent protein kinase catalytic subunit (DNA-PKcs) is indispensable for efficient repair of classical NHEJ, the primary DNA repair pathway for DSBs in human cells, which occur throughout all phases of the cell cycle [27]. Ku specifically binds to DNA DSBs and recruits a repertoire of essential core NHEJ proteins, including DNA-PKcs, X-ray repair cross-complementing protein 4 (XRCC4), ligase IV (LIG4), XRCC4-like factor (XLF), and PAralog of XRCC4 and XLF, also known as C9orf142 or PAXX [ 28, 29]. Upon DNA binding, autophosphorylation of DNA-PKcs induces conformational changes that destabilize the NHEJ core complex, leading to inward sliding of Ku on the DNA and facilitating end-processing and ligase entry into the DNA ends for repair [27]. Autophosphorylation also induces the dissociation of DNA-PKcs from DNA and Ku, thereby rendering the DNA-PK kinase activity inactive [28].

**Figure 2:**
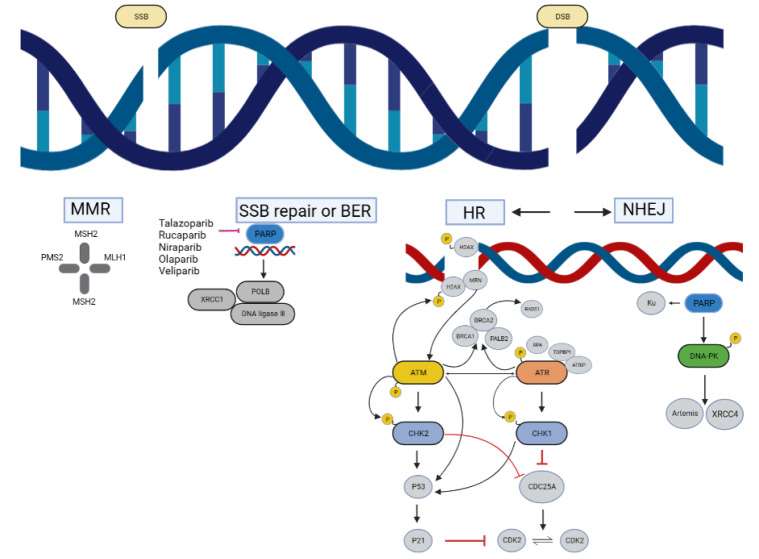
Figure 2 The mechanisms of DDR When double-stranded or single-stranded DNA breaks occur, a series of molecules are recruited at the break sites to facilitate DNA damage repair.

Ataxia telangiectasia mutated (ATM) is the primary kinase responsible for the phosphorylation of histone H2AX on serine 139 (γH2AX) [30]. The ATM protein also facilitates the repair of DNA DSBs in cells and exhibits responsiveness to DSBs occurring throughout the cell cycle. Following a DNA DSB event, ATM is primarily activated through its interaction with NBS1, a component of the MRN complex [ 31, 32] ( Figure 2).

## Combined Application of DDR Inhibitors and Radiotherapy

### Limitations of radiotherapy

To date, apart from surgery, the primary modalities employed for rectal cancer treatment have encompassed radiation and systemic chemotherapy, which primarily induce DNA damage in malignant cells. Notably, radiation therapy constitutes approximately 40% of all therapeutic interventions administered to rectal cancer patients [25]. The anticancer effect is attributed to ionizing radiation, which induces the production of oxygen free radicals within the DNA helix or through water ionization, leading to DNA damage by oxygen free radicals [33]. Studies have shown that ionizing radiation of 1 Gy will produce approximately 1000 SSBs and 35 DSBs [ 34, 35]. Currently, with the advancement of treatment modalities and the guidance provided by imaging technology, precise targeting of tumor lesions can be essentially achieved while effectively preserving normal tissue exposure. However, acute and chronic tissue toxicity remains a limiting factor for radiation dose, such as local dermatitis and local tissue fibrosis, especially radiation enteritis in rectal cancer patients after radiotherapy [36].

Gaining a deeper understanding of the intrinsic radiosensitivity of tumors is crucial for overcoming the current limitations in radiation therapy. A significant breakthrough would be the discovery of a biomarker that accurately predicts radiosensitivity in patients, enabling clinicians to personalize treatment based on serum biomarkers. This could lead to reduced doses for patients with sensitive tumors or omission of concurrent oral chemotherapy sensitizations. Conversely, predicting radiotherapy resistance can optimize treatment strategies for resistant tumors and minimize unnecessary side effects associated with radiotherapy. Extensive research has been conducted on radiosensitive biomarkers in cancer; however, no definitive correlation has been established thus far.

### DDR inhibitors improve radiotherapy sensitivity

A novel approach to enhance the efficacy of radiotherapy is to combine it with targeted drugs, such as DDR inhibitors, which specifically increase the radiosensitivity of tumor cells rather than affecting all cells [37]. Numerous instances of DDR-targeted drugs combined with radiotherapy have demonstrated preclinical efficacy and are currently undergoing clinical trials [ 38‒ 41].

In the field of radiotherapy resistance in rectal cancer, numerous studies on DDR are currently underway, with a focus on identifying novel targets within DDR pathways. For instance, our investigation has revealed that PR domain zinc finger protein 15 (PRDM15) may contribute to the development of radiotherapy resistance in colorectal cancer [42], but the implementation of subsequent studies targeting it with inhibitors is confronted with numerous challenges.

Currently, our comprehension and application of radiotherapy, chemotherapy, or targeted therapy as individual modalities are well-established; however, there remains a significant knowledge gap regarding the optimal integration of targeted therapy, including DDR inhibitors, with radiotherapy [43]. Crucial issues include determining the optimal dosage of DDR as a radiosensitizer, assessing the potential benefits of targeted drugs following radiation-induced DNA damage, and evaluating whether the radiosensitization effects of DDR drugs remain consistent in the presence of specific genetic defects in DDR molecules.

A recent preclinical study investigating the radiosensitivity of PARP inhibitors in breast cancer 2 (BRCA2)-deficient and BRCA2-overexpressing cells has provided valuable insights into these inquiries [44]. The study concluded that addition of PARP inhibitors 7 h post-radiotherapy was sufficient to induce radiosensitization. Moreover, in *BRCA2*-knockout and BRCA2-overexpressing cell lines, a low-dose of PARP inhibitors demonstrated a greater susceptibility for sensitizing *BRCA2-*knockout cells compared to BRCA2-overexpressing cells [44].

### Research difficulties in the combined application of DDR inhibitors and radiotherapy

The effects of targeted DDR drugs in combination with ionizing radiation on normal tissues necessitate further investigation, elucidating whether the enhanced efficacy of antitumor cells is accompanied by increased toxicity to normal tissues [45]. The challenge of this type of preclinical study lies in the requirement for an immunologically competent host model, as it necessitates the identification of both the specific dose of the DDR inhibitor and the radiation dose [46]. While it is feasible to assess antitumor effects and normal tissue toxicity in separate models, an ideal approach would involve employing an animal model with homogeneous or *in situ* immunoactivity to simultaneously evaluate both aspects within the same model [47]. The availability of models meeting this requirement is indeed limited; however, it offers valuable guidance for the clinical combination use. Clinical trials involving combinations of DDR inhibitors and radiotherapy pose greater challenges, particularly during the dose-escalation phase, which necessitates a prolonged assessment period for chronic radiation-induced toxicity. Typically, a given dose group requires a 3-month follow-up before further dose escalation [9]. Moreover, as radiotherapy is predominantly employed as a preoperative neoadjuvant therapy, the local control of disease does not necessarily reflect the overall survival of patients, which often requires several years for evaluation. These inherent challenges in combining radiotherapy with DDR inhibitor research have resulted in numerous clinical trials being conducted.

## Combined Application of DDR Inhibitors and Immunotherapy

The involvement of cellular DNA repair factors in innate immunity has recently been demonstrated [48]. Multiple lines of evidence provide a biological foundation for the combination of immune checkpoint inhibitors (ICIs) and DDR inhibitors, demonstrating synergistic benefits. Additionally, deficiencies in the DDR pathway may serve as predictive biomarkers for ICI reactivity. The utilization of DDR inhibitors has the potential to augment tumor mutation burden (TMB), thereby promoting neoantigen production and enhancing anticancer T-cell activity. This study supports the correlation between high TMB and dynamic neoantigen renewal with epitope-specific T-cell responses in MMR-deficient tumors treated with ICIs [ 49‒ 51].

The ability of cancer cells to evade immune detection and destruction partly relies on the expression of immunosuppressive proteins on their surfaces. PD-L1 binds to the PD-1 receptor of T cells, thereby impeding their proliferation and suppressing their cytotoxic activity [52]. The cytotoxic T lymphocyte-associated antigen 4 (CTLA-4) is an additional negative regulator of immune effectors exploited by tumor cells. This receptor is expressed on the surface of T cells and interacts with B7 molecules on the surface of antigen-presenting cells, thereby impeding T-cell activation [53]. Immune checkpoint blockade holds potential in facilitating T-cell recognition and elimination of neoantigen-positive tumors, thereby warranting further exploration for harnessing neoantigen formation in cancer immunotherapy. The ability of PARPi and/or other DDR inhibitors to enhance mutational burden and induce neoantigen expression in human tumors remains an area requiring investigation.

## Strategies for the Use of DDR Inhibitors as Anticancer Drugs

The objective of targeting DDRs in cancer is to enhance the vulnerability of cancer cells towards DNA damage induced during the S-phase [54]. Synthetic lethality is the best way to apply DDR inhibitors [ 55‒ 57]; for example, the use of PAPR inhibitors in patients with insufficient HR repair will result in cancer cells being unable to process the S-stage DSBs induced by PARP inhibitors [58]. If the extent of S-phase damage reaches a critical threshold, it can potentially trigger cellular demise through replication stress or apoptosis induction [59]. If DSB-induced DNA damage persists into mitosis, it can give rise to mitotic errors, thereby triggering apoptotic cell death [60]. This elucidates the enhanced efficacy of taxane chemotherapeutics, which exert their action during mitosis, when combined with platinoid drugs that augment DNA damage in the S-phase. Consequently, the reliance of S-stage cancer cells on G2/M checkpoint proteins is amplified during DNA damage. Notably, even in the presence of intact G2/M checkpoint proteins, excessive DNA damage can surpass the survival threshold of cancer cells [61]. Therefore, inhibitors targeting G2/M checkpoint proteins such as CHK1 and WEE1 can be efficacious by overriding the cancer cell’s defense mechanism at the G2/M checkpoint [62]. The clinical use of these drugs in conjunction with carboplatin, irinotecan, and other chemotherapeutic agents has been observed, wherein the selection criteria for rectal cancer primarily focus on P53 defects [63] ( Table 1).

**Table:** **Table 1** Representative DDR inhibitors and their mechanisms

Target	Mechanism	Agents
PARP	Initial sensor to mediate the early recruitment of DNA damage repair factors	Olaparib, Veliparib, Talazoparib, Rucaparib, Niraparib
ATM/ATR	Sensor of DSB/chromatin alterations and a signal transducer; sensor of blockage of replication and transcription	AZD0156, VX-970, AZD6738
DNA-PKcs	DNA-dependent protein kinase; NHEJ; DNA damage signaling	NU7441, NU7026, M3814
CHK1/2	Activated by ATR in the case of replication stress to induce intra-S and G2/M cell cycle arrests	LY2603618, SRA-737, GDC0575, AZD7762
WEE1	Inhibits CDKl by phosphorylating it on two different sites to regulate cell cycle progression	AZD1775

In summary, the primary objective of utilizing DDR inhibitors in cancer therapy should be to optimize G1 and S phase DNA damage while impeding G2 phase repair at its most fundamental level [64]. Subsequently, this DNA damage can be transmitted into mitosis, where its consequences can be catastrophic [65]. The results of a recently published clinical trial provide an excellent illustration of this principle. The findings demonstrated an aberrant response in patients with metastatic small cell bladder cancer when treated with a combination of a CHK1/CHK2 inhibitor and irinotecan [ 66, 67]. Another example is RAD50, a protein involved in the DDR pathway that detects DSBs and subsequently activates ATM to initiate DSB repair. Inhibitors targeting RAD50 can be utilized in colorectal cancer patients harboring P53 mutations. Due to the synergistic effect of P53 mutations, DDR inhibitors and chemotherapy, this combination therapy exhibits superior efficacy in eradicating the cancerous tissue [68].

## Review and Progress of DDR Inhibitors in Cancer Therapy

### Review of early DDR inhibitors

The initial strategy for utilizing DDR inhibitors in cancer therapy involves their combination with chemotherapy [69]. The combination of an MGMT [O6-methylguanine (O6meG)-DNA methyltransferase] inhibitor and semustine in early clinical trials yielded limited clinical efficacy and exhibited poor tolerability as a combined treatment option [ 34, 70]. The combination of a PARP inhibitor with temozolomide in metastatic melanoma was initiated in 2003 without selecting any gene mutation to enhance tumor specificity, thereby precluding subsequent phase III trials [71]. The noteworthy aspect lies in the research and application of PARP inhibitors, particularly their synthetic lethality in the context of BRCA1/BRCA2 defects. The efficacy of olaparib monotherapy in cancers harboring BRCA1/BRCA2 mutations underscores the significance of leveraging known DDR deficiencies to elicit distinct effects between malignant and healthy tissues.

### Current status of clinical trials involving DDR inhibitors

In the past decade, there has been a significant increase in the production and clinical development of DDR inhibitors. Clinical trials are currently underway to investigate the efficacy of Chk1/Chk2 and WEE1 inhibitors in combination with chemotherapy for eliminating G2/M checkpoints [62]. We anticipate an imminent surge in clinical trials investigating DDR inhibitors as standalone therapies tailored to specific genetic contexts.

While the majority of DDRs are employed in combination, comprehending their genetic contexts wherein they manifest singular therapeutic benefits is crucial for devising optimal treatment combinations [ 72, 73]. The combination of two DDR inhibitors is also expected to exhibit a more efficacious response in comparison to monotherapy with DDR inhibitors. An illustrative instance involves the synergistic utilization of a PARP inhibitor and a WEE1 inhibitor, supported by substantial preclinical evidence [74]. The potential of combining DDR inhibitors with targeted agents is exemplified by the recently announced phase II trial of olaparib in conjunction with a vascular endothelial growth factor (VEGF) inhibitor [75]. The combination of DDR inhibitors and VEGF inhibitors demonstrated superior efficacy compared to standard platinum-based therapy in wild-type tumors within this study, suggesting the potential for DDR-based therapy to replace more toxic chemotherapy in early treatment and thereby enhance the quality of life for cancer patients. Additionally, investigations have explored the synergistic effects of DDR inhibitors with epigenetic drugs, which modulate the expressions of DDR-related genes in cancer [ 76, 77]. These studies suggest that the combination of epigenetic drugs with DDR drugs can potentiate their effects. For instance, inhibition of histone lysine methyltransferase (HKMT) has been demonstrated to prevent the retention of the BRCA1/BARD1 complex on DNA DSBs, thereby promoting NHEJ and consequently augmenting the efficacy of PARP inhibitors [77]. The numerous associations between DDR and the immune response suggest that combining DDR inhibitors with immunotherapy could potentially revolutionize cancer treatment. Currently, immunotherapy has demonstrated remarkable efficacy but is only utilized in a relatively small subset of patients. Preliminary studies have indicated an association between radiotherapy and anti-ctLA4 [78] as well as anti-PD-L1 therapies [51]. The combination of immunotherapy enhances tumor response. The concurrent administration of DDRs and immunotherapy agents holds promise for augmenting response in cancer patients, with ongoing clinical trials investigating the synergistic effects of PARP inhibitors and immunotherapy.

## Conclusions and Perspects

Long-term data from clinical trials of various PARP inhibitors demonstrate a sustained response primarily in cancer patients with BRCA1/2 mutations, albeit the majority of patients inevitably develop resistance to platinum-based and/or PARP inhibitor therapy [79]. In clinical and preclinical studies, resistance to PARP inhibitors arises through three overarching mechanisms: acquisition of aberrations that enhance homologous recombination repair capacity; activation of signaling pathways to curtail cell cycle progression and mitigate replication stress; and miscellaneous alterations in single DNA damage response pathways that currently defy categorization [80]. Currently, implementation of a synthetic lethal protocol represents an optimal strategy for applying DDR inhibitors in cancer research. Therefore, the key principle for utilizing DDR inhibitors in rectal cancer treatment lies in identifying associated genetic defects specific to this type of cancer. The clinical success of DDR inhibitors is inevitable and the approval of olaparib as the first tumor-specific DDR inhibitor in cancer therapy marks a promising beginning for their significant role in future advancements.
